# Selenized Chickpea Sprouts Hydrolysates as a Potential Anti-Aging Ingredient

**DOI:** 10.3390/molecules28083402

**Published:** 2023-04-12

**Authors:** Sayra N. Serrano-Sandoval, Antonio Jiménez-Rodríguez, Jesús Hernández-Pérez, Rocio Alejandra Chavez-Santoscoy, Daniela Guardado-Félix, Marilena Antunes-Ricardo

**Affiliations:** 1 Escuela de Ingeniería y Ciencias, Tecnologico de Monterrey, Av. Eugenio Garza Sada 2501 Sur, Monterrey 64849, NL, Mexico; 2 The Institute for Obesity Research, Tecnologico de Monterrey, Av. Eugenio Garza Sada 2501 Sur, Monterrey 64849, NL, Mexico; 3 Programa Regional de Posgrado en Biotecnología, Facultad de Ciencias Químico Biológicas, Universidad Autónoma de Sinaloa, FCQB-UAS, AP 1354, Culiacan 80000, SIN, Mexico

**Keywords:** chickpea proteins, protein hydrolysates, skin aging, cosmeceutical effect, collagen, elastase, oxidative stress

## Abstract

Skin aging represents a health and aesthetic problem that could result in infections and skin diseases. Bioactive peptides can potentially be used in skin aging regulation. Chickpea (*Cicer arietinum* L.) selenoproteins were obtained from germination with 2 mg Na_2_SeO_3_/100 g of seeds for 2 days. Alcalase, pepsin, and trypsin were used as hydrolyzers, and a membrane < 10 kDa was used to fractionate the hydrolysate. Se content, antioxidant capacity, elastase and collagen inhibition, functional stability, and preventative capacity were analyzed. Significant increases in Se content were found in germinated chickpea flour and protein related to the control. An increase of 38% in protein was observed in the selenized flour related to the control. A band (600–550 cm^−1^) observed in the selenized hydrolysates suggested the insertion of Se into the protein. Hydrolysates from pepsin and trypsin had the highest antioxidant potential. Se enhanced the stability of total protein and protein hydrolysates through time and increased their antioxidant capacity. Hydrolysates > 10 kDa had higher elastase and collagenase inhibition than the total protein and hydrolysates < 10 kDa. Protein hydrolysates < 10 kDa 6 h before UVA radiation had the highest inhibition of collagen degradation. Selenized protein hydrolysates showed promising antioxidant effects that could be related to skin anti-aging effects.

## 1. Introduction

Bioactive peptides are protein hydrolysates that have shown health-related effects on health that hold great promise as valuable functional ingredients for humans [1]. These hydrolysates range in size from 2 to 50 amino acids and have molecular masses of less than 6000 daltons (Da). Their structural properties can induce beneficial biological activities, such as anti-hypertensive, antioxidative, immunomodulatory, anticancer, antimicrobial, and blood lipid-lowering effects [2,3,4,5,6]. Furthermore, these peptides have demonstrated high biological activity and specificity with a low risk of side effects, making them interesting for the pharmaceutical and cosmetic industries [7].

Bioactive peptides can be naturally generated by digestive proteolysis in the gastrointestinal tract, chemical or enzymatic hydrolysis in vitro, bacterial fermentation, and chemical synthesis [8]. Peptides from plant sources usually include cereals, typically cereals (wheat, barley, corn, rice), pseudocereals (buckwheat and amaranth), pulses (soybean, chickpea, beans, peas, and lentils), and others [9,10,11]. Chickpea is the third-most important pulse crop produced in the world, with a global production of 11.67 million tons annually 12. The main producer countries are India, Turkey, the Russian Federation, Myanmar, Pakistan, Ethiopia, the United States, Australia, Canada, and Mexico [12].

Several studies have reported the biological properties of chickpeas, including antioxidant, anti-hypertensive, hypocholesterolemic, anti-inflammatory, chemopreventive, and healthy gut microbiome promoter [13,14,15]. In recent years, selenization has emerged as a strategy to improve these biological properties, with a special focus on antioxidant activity to modulate oxidative stress, which has been closely related to the development of other chronic degenerative diseases [16]. Selenium (Se) biofortification has shown increases in phenolics content, as well as the formation of selenized proteins and peptides [17,18]. Peptides obtained from germinated chickpeas have shown a selective Se accumulation with a selective increase in antioxidant potential [18].

Considering the antioxidant properties of selenized hydrolysates from chickpeas, as well as the evidence that these compounds can modulate the biological response to oxidative stress processes that involve inflammation and protein degradation, it is important to explore new applications related to skin diseases through the evaluation of their cosmeceutical properties. The cosmeceutical potential of selenized protein and hydrolysates from germinated Kabuli type chickpea (*Cicer arietinum* L.) was evaluated in this study by the determination of their effect on skin oxidative stress, as well as their effect on elastin and collagen using an in vitro model.

## 2. Results

### 2.1. Protein and Selenium Content in Chickpea Sprouts Flour

Significant increments of total Se content by 35- and 18-fold were found in flour and protein extract related to the control, respectively (Table 1). These results agree with those previously reported by [18], who also observed a Se increment of 17–29-fold in the protein extract of chickpea flour that was germinated in the presence of 1 mg and 2 mg of Na_2_SeO_3_/100 g. Moreover, it was observed that total Se content was diminished during the protein extraction method by 35.89% and 67.85% in the control and Se-enriched samples, respectively. It was observed that protein extracts from Se-enriched chickpea sprout flour contained significantly (38.13%) more protein related to the control (Table 1).

### 2.2. Protein Structure by FTIR Spectroscopy

**Figure 1:**
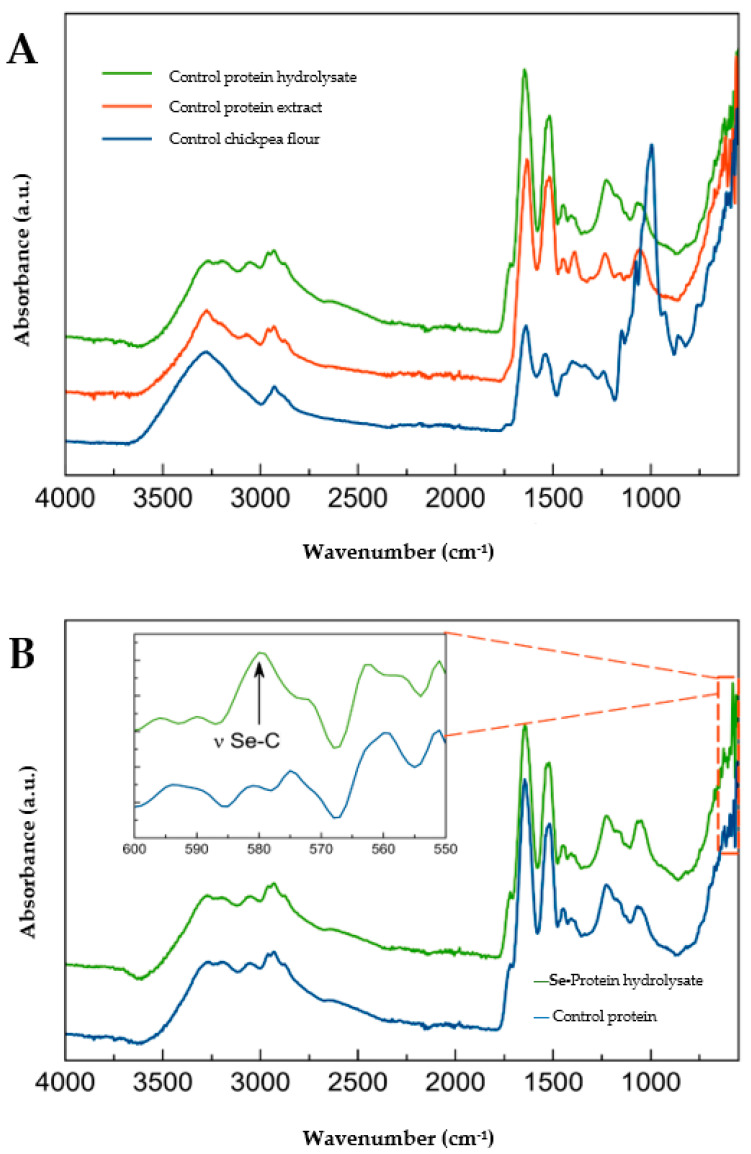
Fourier transform-infrared spectroscopy (FTIR) spectra of (**A**) Control protein hydrolysate with pepsin, protein extract, and germinated chickpea flour; (**B**) Selenized protein hydrolysate with pepsin and control protein hydrolysate.

The spectral intensity of flours, protein extracts, and protein hydrolysates is shown in Figure 1. The chemical changes during the process of protein extraction and enzymatic hydrolysis were analyzed (Figure 1A). The loss of a broad band around 3450 cm^−1^ can be seen. Moreover, bands around 1000 cm^−1^ can be observed, which are absent in the protein extracts. Bands with a larger intensity are shown at 1640 and 1520 cm^−1^.

### 2.3. Enzymatic Hydrolysis and Screening of Antioxidant Activity of Selenized Protein Hydrolysates

The degree of hydrolysis (DH) performed by the enzymes in the selenized protein fraction did not increase significantly during the four instances (30, 60, 120, and 180 min) of enzymatic reaction. The protein hydrolysates with alcalase reached a range of 16.27 to 17.72% DH without significant differences at all hydrolysis times. The highest DH (%) was reached by the enzymatic action of pepsin with a range of 34.43% to 37.56% without significant differences. Finally, trypsin without any significant differences reached 14.06% during the four instances that enzymatic reaction was measured (Table 2).

Scavenging for DPPH radical and oxygen radical absorbance capacity (ORAC), hydroxyl radical (·OH) scavenging activity, superoxide scavenging radical activity, and cellular antioxidant activity (CAA) in HDFa cells of the selenized hydrolysates (25 µg protein/mL) with the different hydrolysis enzyme and times are shown in Table 3. The selenized hydrolysates produced with pepsin showed the highest DPPH scavenging activity related to other hydrolysates; however, no significant differences were found among times (*p* > 0.05). The antioxidant activities of DPPH radical scavenging of the hydrolysates produced at 120 min of hydrolysis with pepsin (14.36 ± 2.13) and those produced with trypsin at 180 min (3.10 ± 1.38) were significantly different (*p* < 0.05). The selenized hydrolysates produced with alcalase at 30 and 60 min showed the highest ORAC scavenging activity and significant differences (*p* < 0.05) related to those produced at 120 and 180 min. The highest ORAC scavenging activity of selenized hydrolysates produced with pepsin was those obtained at 120 min with significant differences among the other times (*p* < 0.05). Overall, the highest ORAC activity was observed in the selenized hydrolysates produced at 120 min with pepsin (98.81 ± 2.67) with an increase of up to 85% which was significantly different (*p* < 0.05) to those produced with alcalase and pepsin at 120 min and those from trypsin at 180 min. In the OH scavenging activity assay, selenized hydrolysates produced with alcalase had no significant differences among times. Furthermore, it was the enzyme that produced hydrolysates with lower antioxidant activity at the four hydrolysis instances. Pepsin and trypsin enzymes produced selenized hydrolysates that still have the highest activities. Selenized hydrolysates produced with trypsin at 30 min of hydrolysis had the highest OH scavenging activity, increasing their antioxidant activity by almost 35% in comparison to the control.

The superoxide scavenging activity showed similarities to previous assays. The selenized hydrolysates from the alcalase enzymatic reaction maintained the lowest activities with no significant differences among times. On the other hand, pepsin hydrolysates produced at 60 min obtained the highest superoxide scavenging activity with significant differences with those from the same enzymatic reaction stopped at 120 and 180 min. Selenized hydrolysates from trypsin action at 60, 120, and 180 min presented the highest superoxide scavenging activity without significant differences. Conversely, the hydrolysates produced from trypsin at 180 min were almost 78% higher and significantly different (*p* < 0.05) from those produced at 30 min by the same enzyme. Selenized hydrolysates produced by alcalase at 30 min presented the highest CAA in HDFa cells with increases up to 3.39-fold and significant differences related to the other hydrolysis times. Pepsin activity at 180 min produced the selenized hydrolysates with higher CAA with significant differences (*p* < 0.05) to those produced at 30 and 60 min. Selenized hydrolysates from trypsin activity at 30 min showed the highest CAA with almost 5.7-fold related to the other hydrolysis times.

### 2.4. Selenized Hydrolysates Stability through Time

The stability of selenized hydrolysates (25 µg protein/mL) was assessed by evaluating their antioxidant activity by superoxide scavenging and cellular antioxidant activity (CAA) in human dermal fibroblasts (HDFa) for 42 days.

**Figure 2:**
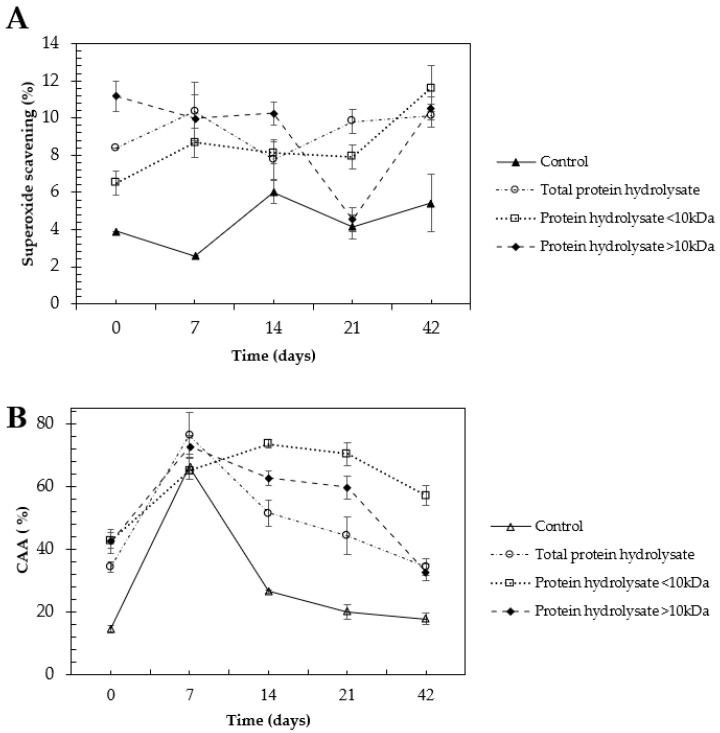
Stability of selenized hydrolysates from chickpea using (**A**) superoxide radical scavenging activity throughout 28 days of storage at −20 °C and (**B**) cellular antioxidant activity (CAA) at 25 µg/mL throughout 42 days of storage at −20 °C. Total selenized hydrolysate, selenized hydrolysate fraction > 10 kDa, selenized hydrolysate fraction < 10 kDa, and total control hydrolysate. Graphs show the means ± standard deviation of a sample of three replicates. The significantly different means are labeled with an asterisk each time the test is evaluated by Fisher´s post hoc test with 95% confidence compared to all days.

There were no significant differences in the superoxide radical scavenging activity throughout the 21 days for most of the treatments. Total selenized protein hydrolysate, selenized hydrolysate fraction < 10 kDa, and selenized hydrolysate fraction > 10 kDa, presented a higher antioxidant activity than the control protein hydrolysate despite the storage time (Figure 2A). Similarly, the total selenized hydrolysate and the selenized hydrolysate fractions (<10 kDa, >10 kDa) were significantly higher in the CAA than the control protein hydrolysate (Figure 2B).

### 2.5. Cosmeceutical Effect of Selenized Hydrolysates

Cell viability was performed in the HDFa cell line to provide an approximate effect of the toxicity of the hydrolysates on the skin using the same previous range of concentrations (0.39 to 100 µg/mL) and assure safe concentrations. Concentrations from 3.12 to 25 µg/mL presented cell viability > 80% and they were used in the following assays.

**Figure 3:**
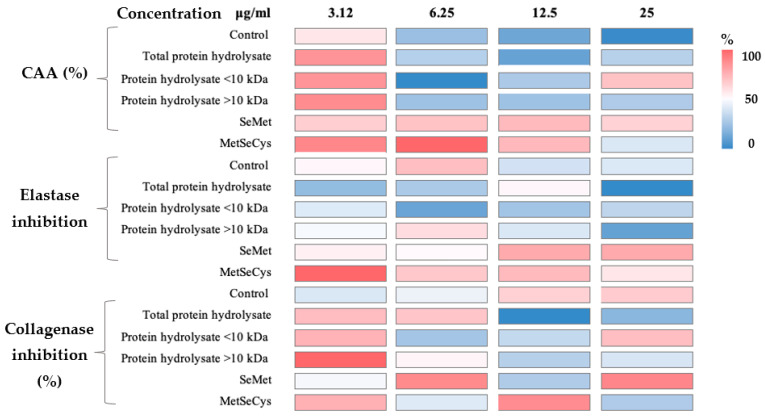
Heat map of the cellular antioxidant activity (CAA) in HDFa cells, elastase inhibition (%), and collagenase inhibition (%) in a range of concentrations from 25 to 3.12 µg/mL of each sample. The complete table is presented in the Supplementary material Table S1. Colors represent the % of the activity of inhibition compared by assays. SeMet: selenomethionine, MetSeCys: methyl selenocysteine.

Cellular antioxidant activity assay (Figure 3, Table S1) showed that standard methyl selenocysteine (MeSeCys) at 6.25 µg/mL exhibited the highest antioxidant activity (76.09 ± 3.3%) without significant differences within the same sample at 3.12 µg/mL. There were no significant differences among the MeSeCys at 3.12 µg/mL related to the total protein hydrolysate, protein hydrolysate < 10 kDa, and >10 kDa at the same concentration. It is important to highlight that although in most of the samples the CAA decreased at higher concentrations, the selenized hydrolysates resulted in most of the treatments being higher than the control.

Regarding elastase inhibition (Figure 3), the significantly highest elastase inhibition was exerted by the MetSeCys at 3.12 µg/mL (27.58 ± 2.75) related to the other concentrations and assays. Followed by elastase inhibition exerted by the standard selenomethionine (SeMet) at 12.5 and 25 µg/mL and MetSeCys at 12.5 µg/mL without significant differences (*p* > 0.05). The control and selenized protein hydrolysate > 10 kDa exerted their highest activity at 6.25 µg/mL (19.05 ± 1.17 and 16.02 ± 0.74%, respectively) and the total protein hydrolysates did so at 12.5 µg/mL (13.32 ± 4.31%). The highest activity that selenized hydrolysate < 10 kDa achieved was almost half (10.76 ± 0.02%) that of MetSeCys at the same concentration.

The highest collagenase inhibition was obtained by the selenized hydrolysates > 10 kDa with 97.44 ± 2.30%. Increases of 43.3 and 46.7% were found in the control at 12.5 and 25 µg/mL, respectively, related to the lowest concentration. Moreover, the highest activity of total protein hydrolysates, protein hydrolysates < and >10 kDa were found at 3.12 µg/mL (74.78 ± 1.65, 77.73 ± 4.05, and 97.44 ± 2.30%, respectively). On the other hand, the highest activity of SeMet was found in the samples at 25 µg/mL (89.55 ± 15.05%) with an increase of 62.7% related to the lowest concentration.

During the photoaging test, type I collagen concentration and production of reactive oxygen species (ROS) were measured in fibroblasts (HDFa cells) after treatment with sprouted chickpea hydrolysates for 6 h before UVA radiation. As can be seen in Table 4 a four-fold reduction in type I collagen degradation was found in the HDFa cells treated with hydrolysates < 10 kDa 6 h before radiation related to the control. The lowest ROS production was found in SeMet and MeSeCys which could be related to their highest type I collagen concentration.

## 3. Discussion

### 3.1. Protein and Selenium Content in Chickpea Sprouts Flour

Total Se content was diminished during the protein extraction method in the control and Se-enriched samples, which could be related to the protein content in the samples of 0 and 2 mg Na_2_SeO_3_/100 g, which contained only 33.41 ± 5.87 and 46.15 ± 6.79% of the total protein, respectively. In addition, [19] reported that Se is normally found in protein that is not able to be extracted from the control flour and bound in starch complexes.

The higher protein content from Se-enriched chickpea sprout flour could be demonstrated because the sulfur and nitrogen metabolic pathways in plants are affected by Se assimilation. Se-induced changes in sulfur assimilation might influence nitrogen metabolism, including protein and amino acid synthesis. The increment of the overall protein and amino acid content of plants, caused by Se accumulation at a proper dose might improve their nutritional value [20].

### 3.2. Protein Structure by FTIR Spectroscopy

The main differences are the loss of a broad band around 3450 cm^−1^ that is related to -OH vibrations of carbohydrate groups, which can be related to starch and other sugar components of the chickpea flour. Moreover, the bands around 1000 cm^−1^ from the flour may be related to stretching of the C-C, C-O, and C-OH bonds of starch [21], which are not present in protein extracts due to the extraction process where carbohydrates are removed. This process of protein purification can be observed at bands of 1640 and 1520 cm^−1^ which have a larger intensity of protein extracts and hydrolysates, proving that proteins are concentrated from carbohydrates structures such as starch.

According to [22], there are great similarities in the infrared spectra of Se compounds and their sulfur analogs. However, little changes such as change in mass, weaker bonds linked to Se and slight variation in the bond angles account for the spectral differences. The appearance of a band of 600 to 550 cm^−1^ in the hydrolysates from selenized chickpea flour suggests the insertion of the Se atom into the protein sequence (Figure 2A). It is reported that selenides such as C-Se-C exhibit a band among stretching of 610–550 cm^−1^ with weak and medium intensity. In addition, a weak band of stretching in the bond C-Se was reported at 625 to 530 cm^−1^ [22].

### 3.3. Enzymatic Hydrolysis and Screening of Antioxidant Activity of Selenized Protein Hydrolysates

The difference in DH (%), and thus antioxidant activities may be related to the amino acid content of selenized protein from germinated chickpeas and the sequence where the enzyme cleaves. Alcalase is an endopeptidase that cleaves peptide bonds from non-terminal amino acids randomly, thereby facilitating further hydrolysis of proteins [23]. On the other hand, pepsin is an acid protease that preferentially cleaves on the C-terminal end of Met, Phe, and Leu, although it is not very specific [24]. Finally, trypsin is a member of the S1 family of serine endopeptidases, which cleaves peptide bonds on the C-terminal side of lysine and arginine residues. Peptide bonds at the C-terminal of Arg are generally cleaved faster (2- to 10-fold) than at Lys residues [25]. Thus, the higher DH (%) for pepsin may be related to the high content of Met, Phe, and Leu amino acids in the protein extract and higher antioxidant activities. Furthermore, the activities of bioactive peptides are governed by peptide structural features, such as amino acid properties, C- and N-terminal amino acid types, peptide chain length and weight, and hydrophobicity. The presence of hydrophobic and aromatic amino acid residues in peptides, particularly His, Pro, Tyr, Phe, Leu, Ile, and Ala, are correlated to their radical-scavenging capabilities [20]. This behavior agrees with pepsin hydrolysates, in which the enzymes cleaved at Phe and Leu, demonstrate a high activity in most radical scavenging assays.

After a chemical antioxidant activity screening, it was concluded that pepsin and trypsin had the highest antioxidant potential to have a higher cosmeceutical effect. In addition, knowing that between these two hydrolysates, there is no significant (*p* > 0.05) difference, the pepsin hydrolysate obtained at 60 min was chosen for further analysis because this represents lower time and energy consumption for further industrial production.

### 3.4. Selenized Hydrolysates Stability through Time

It has been demonstrated that the pattern or position of disulfide bonds contributes significantly to protein folding, decreasing their conformational entropy and thus influencing their stability and biological activity [26,27]. Se and sulfur (S) belong to the same family in the periodic table and occur in proteins as constituents of the amino acids cysteine, methionine, selenocysteine, and selenomethionine. The insertion of Se into proteins makes them ideal catalysts for many biological redox transformations [28,29]. It has been demonstrated that Se-centered hydrogen bonds contribute to the structural stability of proteins, and this could be due to a significant increase in electrostatic and polarization interactions [29]. Some authors investigated the redox-responsive drug release of prodrug nano-assemblies, evaluating the impact of the presence of S, Se, and C bonds in these. Se bond-bridged prodrug nano-assemblies showed more sensitivity to oxidation stimuli than S bond-bridged prodrug nano-assemblies due to the weaker electronegativity of Se derived from its larger atomic radius [30]. Likewise, diselenide bond-bridged prodrug nano-assemblies exhibited the most potent antitumor activity due to their good colloidal stability, long circulation time, high tumor accumulation, and efficient drug release in tumor cells [30].

The highest antioxidant activities in the superoxide scavenging and CAA were found in the selenized hydrolysates fraction < 10 kDa. There is evidence that peptides < 10 kDa exert higher antioxidant properties. An increase of 51% of CAA with a glutelin fraction < 10 kDa supplemented with Se during chickpea germination (2 mg Na_2_SeO_3_/100 g seeds) has been reported [18]. The potential functional peptides in the range of 3–10 kDa from Eleusine coracana protein hydrolysate with the highest antioxidant properties have been reported [31]. Likewise, [32] studied peptides’ fraction from 3 to 20 kDa from germinated amaranth subjected to in vitro simulated gastrointestinal digestion. The authors reported that peptides < 15 kDa from the hydrolysis of protein from chia (*Salvia hispanica* L.) showed the greatest antioxidant activities determined by DPPH and ABTS assays [33].

### 3.5. Cosmeceutical Effect of Selenized Hydrolysates

Some reports showed the high potential of selenized peptides or proteins against oxidants in the in vitro assays. It has been shown that selenized peptides of glutelin < 10 kDa significantly exerted the highest CAA (%), this similar behavior was found in the highest concentration (25 µg/mL) of hydrolysates < 10 kDa [18]. Furthermore, it was reported that Se-rich peptide from yeast protein fraction showed a protective effect against H_2_O_2_-induced cytotoxicity in epidermal keratinocyte (HaCaT) cells, resulting in a promising antioxidant nutrient to minimize skin oxidative damage [34]. Furthermore, it was stated that selenized glutelin exhibited the highest CAA with an increase of 11% compared to the control. Generally, it was observed that a higher concentration of the samples results in lower CAA [35]. It was reported that there is a saturation in the antioxidant activity of peptides from fermented grains (Juipei) that reaches 1000 mg/L, even leading to a decrease in activity [36]. Therefore, it could be deduced that in this study, a higher concentration of the samples resulted in similar or lower antioxidant activity. There is scarce information about the inhibition of elastase and collagenase activities mediated by Se peptides [36]. Information on bioactive peptides that exert cosmeceutical applications has been published. The authors described that those peptides from spirulina and collagen peptides from 1–3 kDa exerted collagenase inhibitory potential. Furthermore, the authors described that collagen and microalgae peptides showed high elastase inhibition [37]. As can be observed, depending on the assay’s different sample concentrations, it is necessary to obtain a positive result. More studies are necessary to elucidate the exact mechanisms by which peptides could improve skin health, avoiding the degradation of the protein matrix of the skin.

According to [38], the main difference can be seen in the collagen proteins in a membrane protein complex in dermal fibroblasts during skin aging, i.e., collagen type I, which accounts for around 80% of the overall collagen in the adult human dermis and its content could indicate degradation. It is shown that Se-enriched tuna enhances collagen synthesis and promotes the proliferation of fibroblasts having anti-aging and anti-wrinkle effects [39]. Furthermore, it is shown that low doses of Se (30 nM) provide potent protection against UVA-induced cytotoxicity in young keratinocytes (from 20–30-year-old donors), while higher concentrations (240 nM) are required for protective efficacy in old keratinocytes (from donors 60–70 years old) [40]. These studies showed that Se supplementation could be a new strategy for combating skin aging and photoaging.

## 4. Materials and Methods

### 4.1. Biological Material

Kabuli chickpea (*Cicer arietinum* L.) seeds, cultivar Blanco Sinaloa, were obtained from Angostura, Sinaloa, Mexico. The biological material was grown and harvested during the 2015 season and was stored at −20 °C to avoid contamination. Cell lines of primary human dermal fibroblasts, adult (HDFa), and mouse macrophages (Raw 264.7) were purchased from American Type Culture Collection (ATCC, Manassas, VA, USA). 

### 4.2. Chemical Reagents

Sodium selenite (Na_2_SeO_3_) (Sigma-Aldrich, St. Louis, MO, USA), sodium hypochlorite (NaOCl) (Sigma-Aldrich, St. Louis, MO, USA). Hexane, sodium hydroxide, and hydrochloric acid (Desarrollo de Especialidades Químicas, Monterrey, Nuevo León, Mexico). Alcalase 2.4 L FG (Novozymes, Gladsaxe, Denmark), porcine pepsin (Sigma-Aldrich, St. Louis, MO, USA), and trypsin from porcine pancreas (Sigma-Aldrich, St. Louis, MO, USA), Pierce TM BCA Protein Assay Kit (Thermo Fisher Scientific, Waltham, MA, USA). 2,2-Diphenyl-1-picrylhydrazyl (DPPH), 1,10-phenanthroline, fluorescein, 2,2′-Azobis(2-methylpropionamidine) dihydrochloride (AAPH), Tris-HCl, Ethylenediaminetetraacetic acid (EDTA), iron (II) chloride, seleno-L-methionine, and Se-(methyl)selenocysteine·HCl (Sigma-Aldrich, St. Louis, MO, USA). Iron (II) sulfate heptahydrate (Productos Químicos de Monterrey, Monterrey, NL), hydrogen peroxide (Laboratorios Jaloma, Guadalajara, Jalisco, Mexico), and pyrogallol (Sigma-Aldrich, St. Louis, MO, USA). Elastin from porcine pancreas Type IV, collagenase from Clostridium histolyticum, MMP-2 substrate (MCA-Pro-Leu-Ala-Nva-DNP-Dap-Ala-Arg-NH2), N-Succinyl-Ala-Ala-Ala-p-nitroanilide, collagen type I from rat tail, and Direct 80 (Sigma-Aldrich, St. Louis, MO, USA). Dulbecco’s Modified Eagle’s Medium (DMEM-F12), phosphate buffer saline (PBS) at pH 7.4, 0.25% Trypsin-EDTA (1×), Penicillin (10,000 Units/mL)-Streptomycin (10,000 µg/mL) solution, and fetal bovine serum (FBS) were purchased from GIBCO^®^/Life Technologies (Grand Island, NY, USA). Cell Titer 96 Aqueous and Griess reagents were obtained from Promega (Madison, WI, USA). In all experiments, Milli-Q water was used.

### 4.3. Germination of Chickpea in Presence of Selenium (Se)

Disinfection, soaking, and germination in the presence or absence of Se were carried out following exactly the methods described by [17]. Briefly, the seeds (100 g) were disinfected with sodium hypochlorite solution (200 mL) and rinsed with distilled water. Then, chickpea seeds were soaked for 6 h in the presence of selenium solution (85 mL of 2 mg Na_2_SeO_3_) (Sigma Aldrich, St. Louis, MO, USA) at room temperature, while the control was soaked with distilled water. Germination was performed in an incubator (24 ± 1 °C and 80% of relative humidity) by watering with 10 mL of distilled water every 12 h. Finally, the germinated chickpeas were stored at −80 °C until used.

### 4.4. Sprouted Chickpea Flour Production

Sprouted chickpeas were placed on a metal tray and dried in a convection oven (European UNOX, Padua, Italy) at 50 ± 1 °C for 24 h. Dried seeds were ground after drying and seeds were milled in a grinder Standard Model no. 5 (Wiley Mill, Swedesboro, NJ, USA) followed by a Cyclone sample mill (UDY Corporation, Fort Collins, CO, USA). After milling, the samples were passed through a mesh sieve no. 60 (250 µm).

### 4.5. Protein Extraction

Total protein extraction from sprouted chickpea flour was performed following exactly the methods described by [18].

### 4.6. Total Selenium Quantification

The total selenium (Se) content (^77^Se) of sprouted chickpea flour previously digested by microwave digestor (Mars 5 CEM, Matthews, NC, USA) was obtained by an inductively coupled plasma mass spectrometer (ICP-MS) × Series 2 (Thermo Fisher Scientific, Waltham, MA, USA) following the method described by [17]. The total Se content was expressed as μg of Se/g of flour in dry weight (DW).

### 4.7. Enzymatic Hydrolysis of Protein Fraction

Protein hydrolysates were obtained following the protocol by [41] with some modifications. Briefly, the protein extract was solubilized in milli-Q water (5% *w*/*v*) and combined with alcalase, pepsin, or trypsin (4% *w*/*w*, protein base). The reaction started when the optimum parameters for the highest activity of each enzyme were set (alcalase: 60 °C, pH 8.0; pepsin: 37 °C, pH 2.2; trypsin: 37 °C, pH 8.0). The pH of the solution was maintained by continuously adding 1 M NaOH and 1 M HCl. Aliquots were taken for times of 30, 60, 120, and 180 min replacing their volume with milli-Q water after adjusting pH. The mixtures were put in a water bath at 90 °C for 5 min to inactive the enzyme and end the reaction. The volumes of acid or base were recorded to calculate the degree of hydrolysis using the pH-stat method [42]:

DH (%) = h/h_tot_ × 100 = [(BN_b_)/(αM_p_h_tot_)] × 100 (1)

where B is the acid/base consumption in mL, N_b_ is the normality of the base, M_p_ is the mass of hydrolyzed protein (g), h_tot_ is the total number of peptide bonds in the protein substrate (6.25 m equiv./g chickpea sprouts protein), and α is the average degree of dissociation of the α-NH_2_ groups released during hydrolysis, expressed as:

1/α = 10^(pK − pH)^ + 1 (2)

where pH and pK are the values at which proteolysis was performed.

### 4.8. Fourier Transform-Infrared Spectroscopy

Control chickpea flour, protein extract and hydrolysates, and selenized protein hydrolysates were analyzed by Fourier-transform infrared spectroscopy (FTIR) using a PerkinElmer Spectrum One spectrometer equipped with an attenuated total reflection (ATR) accessory (PerkinElmer, Norwalk, VA, USA). The spectra were obtained within the wavenumber range of 550–4000 cm^−1^, with a 4 cm^−1^ resolution, scan number of 8, force gauge > 90 Gauge, and absorbance units. The regions of specific interest in this study included the heights of the carbohydrates zone (≈1000 cm^−1^) and protein amide I, II, and III groups.

### 4.9. DPPH Radical Scavenging Assay

DPPH (2, 2 diphenyl-1-picrylhydrazyl) radical scavenging capacity was analyzed by the method of [43] with some modifications. Briefly, 50 µL of protein hydrolysates (25 µg protein/mL) were mixed with 50 µL of 60 µM DPPH (solubilized in methanol) and placed in a 96-well plate and incubated at 37 °C for 1 h. Absorbance was measured at 515 nm using a microplate reader (Synergy HT, Bio-Tek, Winooski, VT, USA) and water as blank. The scavenging capacity was calculated using the following equation:

DPPH Scavenging (%) = [(A_blank_ − A_sample_)/A_blank_] × 100 (3)

### 4.10. Oxygen Radical Absorbance Capacity Assay (ORAC)

ORAC assay was performed following the protocol described by [44] with some modifications. Briefly, 140 μL of protein hydrolysates (25 µg protein/mL) in 75 mM phosphate buffer (PBS) (pH 7.4) was mixed with 70 μL of 200 nM fluorescein and incubated at 37 °C in the dark for 15 min. Then, 70 μL of 80 mM AAPH (2,2′-Azobis(2-methylpropionamidine) dihydrochloride) was added and the fluorescence was monitored for 100 min reading each minute using excitation and emission wavelengths of 485 and 538 nm, respectively. PBS was used as blank. The integration of the relative fluorescence curve was used to calculate the area under the curve (AUC). Results are expressed according to the following equation:

ORAC (%) = [AUC_blank_ − AUC_sample_)/AUC_blank_] × 100 (4)

### 4.11. Hydroxyl Radical (·OH) Scavenging Assay

The hydroxyl radical (·OH) scavenging assay method described by [45] was used with some modifications. Samples (25 µg protein/mL) were diluted in 0.1 M PBS (pH 7.4). The reactions were carried out in a 96-well microplate, mixing 50 μL of samples or buffer (blank), 50 µL of 3 mM 1,10-phenanthroline in PBS (pH 7.4), and 50 µL of 3 mM FeSO_4_. Afterward, 50 µL of 0.01% hydrogen peroxide (H_2_O_2_) was added to initiate the Fenton reaction, and absorbance was read at 536 nm every 10 min for 1 h while incubating at 37 °C, shaking for 10 s before every reading [45]. The percentage of hydroxyl radical scavenging activity was calculated using the following equation:

OH radical scavenging (%) = [(∆A_blank_ − ∆A_sample_)/∆A_blank_] × 100 (5)

where ΔA_blank_ and ΔA_sample_ are the change in absorbance of the reaction of blank and sample from 0 to 1 h, respectively.

### 4.12. Superoxide Radical Assay

The method described by [45] was followed for the superoxide radical scavenging activity assay with some modifications. Briefly, samples (25 µg protein/mL) were prepared in 50 mM Tris-HCl buffer, pH 8.3 containing 1 mM EDTA. In a 96-well microplate, the sample (80 µL) was mixed with 40 µL of 1.5 mM pyrogallol in 10 mM HCl, reading immediately at 420 nm for 4 min at intervals of 1 min. Tris-HCl buffer, pH 8.3 containing 1 mM EDTA was used for the blank. Results are expressed as the following equation.

superoxide scavenging activity (%) = [(∆Amin^−^^1^ (blank) − ∆Amin^−^^1^(sample))/∆Amin^−^^1^ (blank))] × 100 (6)

where ΔAmin^−^^1^ (blank) and ΔAmin^−^^1^ (sample) are the change in the rate of reaction of blank and sample, respectively.

### 4.13. Cell Viability Assay

In a 96-well plate, HDFa cells (2 × 10^4^ cells per well) were seeded in 100 µL Dulbecco’s modified Eagle’s medium (DMEM-F12), supplemented with 10% fetal bovine serum and incubated (37 °C, 5% CO_2_) for 24 h. After incubation, 100 µL of hydrolysates (prepared in culture medium) were added in triplicate and to a sample blank consisting of culture medium and hydrolysates without cells. For control cells, 100 µL of culture medium was added to cells and the control blank. The samples were incubated for 24 h for individual experiments. After that period, 10 µL of CellTiter 96^®^ Aqueous One Solution reagent (Promega) was added to each well and was incubated for 50 min. Then, absorbance was read at 490 nm. The formula for cell viability estimation is as follows:

Cell Viability (%) = [(A_sample_ − A_sample’s blk_)/(A_control_ − A_control’s blk_)] × 100 (7)

where A_control_ is the absorbance of culture medium with cells, and the CellTiter reagent, whereas A_sample_ is the absorbance of hydrolysates, culture medium with cells, and CellTiter reagent. Blanks (blk) contained all the components except the cells.

### 4.14. Cellular Antioxidant Activity Assay

Cellular antioxidant activity (CAA) was evaluated according to [46] with some modifications. Briefly, in a black 96-well plate, HDFa cells (5 × 10^4^ cells per well) were seeded in Dulbecco’s modified Eagle’s medium (DMEM-F12) supplemented with 10% fetal bovine serum and incubated for 24 h at 37 °C and 5% CO_2_. After 24 h cells were rinsed with phosphate buffer solution (PBS) and incubated for 20 min with 100 µL of hydrolysates and 60 µM of dichlorodihydrofluorescein diacetate (DCFH-DA) (1:1, *v*/*v*). Following rewashing, 100 µL of 2,2′-azobis (2-methylpropionamidine) dihydrochloride (AAPH, 100 mM) was added to generate oxidative stress. Fluorescence emitted at 538 nm with 485 nm excitation was monitored every 2 min for 90 min at 37 °C. The positive control cells were cultured with DCFH-DA (without hydrolysates) and stimulated with AAPH. The following formula was used to determine CAA as a percentage:

CAA (%) = 1 − (ʃ SA − ʃ CA)(8)

where ʃ SA is the integrated area of the hydrolysate fluorescence during the 90 min of the reading and ʃ CA is the integrated area of the control.

### 4.15. Cosmeceutical Effect of Selenized Hydrolysates

#### 4.15.1. Protein Fractioning

Protein hydrolysates that showed the highest antioxidant activity were passed through 10 kDa cutoff filters (Centrifugal Filter Units, Amicon™ Ultra-15, Merck Millipore, Burlington, MA, USA). The filtrates were recovered when they were centrifuged (5000× *g*, 30 min), obtaining two fractions: F1 (>10 kDa) and F2 (<10 kDa) besides the raw protein hydrolysate.

#### 4.15.2. Elastase Inhibitory Activity

The method from [47] was followed to evaluate the inhibitory effect of hydrolysates on elastase with some modifications. Elastase from the porcine pancreas and its substrate (N-succinyl-tri-L-alanine-4-nitroanilide) was dissolved in Tris buffer (pH 8.0). Then, 30 μL of the samples were mixed with 10 μL of elastase and 100 μL of Tris buffer in a 96-well plate. Then, the plate was incubated at 25 °C for 20 min. After that time, 40 μL of the substrate was added, and the absorbance was measured at 410 nm for 20 min with reads every 1 min. Water was used as blank. Inhibition of elastase activity was obtained by the following equation:

Elastase inhibition (%) = [(AUC_blank_ − AUC_sample_)/AUC_blank_] × 100 (9)

where AUC_blank_ is the area under the curve of the blank in the first 10 min and AUC_sample_ is the area under the curve of the hydrolysates in the same period.

#### 4.15.3. Collagenase Inhibitory Activity

The collagenase inhibitory activity of the samples was determined using a spectrofluorimetric technique [48] with some modifications. Briefly, 25 µL of Tris-HCl buffer (10 mM, pH = 7.3), 25 µL of the sample (dissolved in Tris-HCl buffer), and 25 µL of collagenase from C. histolyticum (100 µg/mL in Tris-HCl buffer) were preincubated for 10 min at 37 °C in a 96-well microwell plate. After that, 25 µL of 50 µM MMP-2 substrate solution (MCA-Pro-Leu-Ala-Nva-DNPDap-Ala-Arg-NH2) was added to the buffer. After 30 min of incubation (in dark conditions) at 37 °C, fluorescence levels were measured at an excitation wavelength of 320 nm and an emission wavelength of 405 nm. The inhibition was calculated considering the following equation:

Inhibition (%) = [(F_control_ − F_control’s blk_) − (F_sample_ − F_sample’s blk_)/(F_control_ − F_control’s blk_)] × 100 (10)

where F_control_ is the fluorescence of buffer, collagenase, sample solvent, and substrate and F_sample_ is the absorbance of buffer, collagenase, extract, and substrate. Blanks (blk) contained all the components except the enzyme.

#### 4.15.4. Anti-Photoaging Activity

For the in vitro evaluation of anti-photoaging activity, the method reported by [49] was used incorporating some modifications. Human fibroblast (HDFa) cells were seeded (2 × 10^4^ cells per well) in a medium with FBS and they were incubated for 24 h. After that time, the medium was discarded and cells were washed with PBS twice, replacing the medium without FBS and adding the hydrolysates (prepared in DMEM-F12) at 3.125 µg/mL of concentration for 6 h during incubation to observe their preventive effect against photoaging. At the end of that period, DMEM-F12 was discarded, and cells were washed twice with PBS to then leave a thin layer of PBS. The microplate was placed 5 cm under a UV lamp (model UVGL-55, UVP Inc., Upland CA, USA) for a dose of 5 J cm^−2^ of UVA radiation (365 nm) for 30 min. After irradiation, PBS was discarded, and the cells were incubated with DMEM-F12 for 24 h until the following two irradiations. Before the third irradiation, the production of reactive oxygen species (ROS) was determined by incorporating DCFH-DA into DMEM-F12 and incubating for 20 min before stressing the cells with UVA radiation and observing oxidative stress by fluorescence as previously mentioned in the CAA assay (Section 4.14). After this irradiation, cells were incubated for 24 h to allow fibroblasts to recover from external stress. The cells were kept for collagen determination.

#### 4.15.5. Collagen Quantification

Determination of collagen content was conducted using the colorimetric method described by [50] with slight modifications. HDFa cells (2 × 10^4^ cells per well) were washed twice with PBS and further fixated with cold methanol (−20 °C) for 10 min. Methanol was discarded and cells were treated with 0.1% (*w*/*v*) Sirius Red (Direct Red 80) in saturated picric acid and stained for 1 h at room temperature. The remaining solution was discarded, and cells are washed with PBS at least two times or until there was no picrosirius solution. The collagen-bound stain was dissolved with 0.1 M NaOH for 5 min to then read absorbance at 540 nm. A collagen type I standard curve (0.15–50 µg/mL) was performed by staining collagen standard with picrosirius solution, then centrifuged at 4000× *g* for 10 min, and the pellet was solubilized in 0.1 M NaOH [51].

### 4.16. Statistical Analysis

The independent variables of this experiment were the antioxidant capacity by DPPH, ORAC, hydroxyl radical scavenging, superoxide radical scavenging, iron chelation, CAA, collagenase and elastase inhibition, and collagen content. The dependent variables were the type of enzyme used, the hydrolysate concentration, the time of hydrolysis, and the type of radiation. All experiments were performed in triplicate and the results were expressed as mean with standard deviation. JMP-Software and Minitab Software were used to construct graphs and perform ANOVA followed by Tukey’s or Fisher’s post hoc tests with a 95% confidence level.

## 5. Conclusions

The cosmeceutical potential of the selenized protein and hydrolysates obtained from the selenized chickpea sprout flour was evaluated in this study. Total Se content increased by up to 35- and 18-fold in flour and protein extract related to the control. Likewise, Se influenced the total protein, and an increment of 38.13% was found in the selenized protein extracts related to the control. The appearance of a band of 600 to 550 cm^−1^ in the selenized hydrolysates could confirm the insertion of Se atom into the protein sequence. The hydrolysates from pepsin and trypsin enzymes exerted the highest antioxidant potential. Furthermore, Se enhanced the stability of total protein and protein hydrolysates through time related to the control. The highest CAA (%) was observed in the hydrolysates at 3 µg/mL. The hydrolysates > 10 kDa had higher % elastase and collagenase inhibition than the total protein and hydrolysates < 10 kDa. A treatment of 3.125 µg/mL of protein hydrolysates < 10 kDa 6 h before UVA radiation reduced the degradation of type I collagen by 301% related to the control. Although the results indicate that selenized hydrolysates have anti-aging potential, there are some limitations to these results. The experiments were carried out using 2D in vitro models, knowing that skin is a complex system, in vivo models are required to have a better understanding of what mechanisms could be playing an important role in the anti-aging process of selenized hydrolysates. Furthermore, this is the first step in the research of selenized hydrolysates and their potential use in cosmetics; more experiments are required to know how to introduce and stabilize these hydrolysates in a functional formulation for skin.

## Figures and Tables

**Table 1:** Total selenium (Se) content in germinated chickpea flour and protein extract.

Source	mg Na_2_SeO_3_/100 g	Se Content (mg/kg Sample)
Germinated chickpea flour	0	0.78 ± 0.12 ^c^
	2	27.50 ± 1.98 ^a^
Protein extract	0	0.50 ± 0.00 ^c^
	2	8.84 ± 1.65 ^b^

Means ± standard deviation of 50 samples. Significant different means are labeled with different letters in each column according to Fisher’s test post hoc with 95% of confidence.

**Table 2:** Degree of hydrolysis (%) of alcalase, pepsin, and trypsin at 30, 60, 120, and 180 min.

	Degree of Hydrolysis (%)			
Enzyme	30 min	60 min	120 min	180 min
Alcalase	14.29 ± 2.81 ^b^	17.18 ± 0.26 ^b^	18.63 ± 1.28 ^b^	18.63 ± 1.28 ^b^
Pepsin	31.30 ± 4.43 ^a^	33.49 ± 5.75 ^a^	36.12 ± 2.04 ^a^	37.12 ± 0.63 ^a^
Trypsin	11.50 ± 3.62 ^b^	13.10 ± 1.36 ^b^	13.52 ± 0.77 ^b^	14.48 ± 0.59 ^b^

The degree of hydrolysis was performed with the following conditions temperature and pH. Alcalase 60 °C and pH 8, pepsin 37 °C and pH 2.2, and trypsin 60 °C and pH 8. Data are expressed as means ± standard deviation (*n* = 2). ^a,b^ Significant different means are labeled with different letters in each column of the graph evaluated by Tukey’s test post hoc with 95% confidence compared to all types of cleavage (enzymes and times).

**Table 3:** Screening of antioxidant capacity of selenized hydrolysates cleavage with different enzymes (alcalase, pepsin, and trypsin) and times (30, 60, 120, and 180 min) of selenized hydrolysates from germinated chickpea at 25 µg/mL.

		Antioxidant Activity (%)		
	Hydrolisis Time (min)	Alcalase	Pepsin	Trypsin
DPPH scavenging	30	3.59 ± 0.69 ^bc^	13.62 ± 2.42 ^abc^	13.38 ± 1.38 ^abc^
	60	12.40 ± 1.96 ^abc^	14.85 ± 6.23 ^ab^	7.01 ± 8.31 ^abc^
	120	7.99 ± 0.00 ^abc^	14.36 ± 2.13 ^a^	9.95 ± 0.00 ^abc^
	180	8.73 ± 3.81 ^abc^	12.40 ± 2.77 ^abc^	3.10 ± 1.38 ^c^
ORAC (%)	30	78.27 ± 0.42 ^de^	84.42 ± 9.40 ^cd^	95.93 ± 0.00 ^ab^
	60	69.19 ± 4.42 ^e^	81.89 ± 3.72 ^cd^	90.82 ± 0.00 ^abc^
	120	53.46 ± 7.16 ^f^	98.81 ± 2.67 ^a^	86.70 ± 0.00 ^bcd^
	180	57.92 ± 3.93 ^f^	88.19 ± 1.68 ^bc^	84.82 ± 0.00 ^cd^
OH scavenging (%)	30	11.65 ± 1.50 ^d^	17.64 ± 2.29 ^abc^	22.63 ± 1.73 ^a^
	60	14.14 ± 1.32 ^cd^	17.80 ± 1.52 ^abc^	21.63 ± 1.41 ^ab^
	120	11.31 ± 0.76 ^d^	21.63 ± 3.53 ^ab^	18.30 ± 2.02 ^abc^
	180	13.14 ± 0.00 ^cd^	21.63 ± 2.12 ^ab^	17.39 ± 2.47 ^bcd^
Superoxide scavenging (%)	30	5.41 ± 0.00 ^e^	14.97 ± 1.35 ^ab^	9.24 ± 0.00 ^cde^
	60	7.80 ± 0.68 ^de^	18.31 ± 0.68 ^a^	14.01 ± 0.00 ^abc^
	120	7.32 ± 1.35 ^de^	6.37 ± 2.70 ^e^	11.62 ± 0.00 ^bcd^
	180	7.32 ± 0.00 ^de^	6.37 ± 2.70 ^e^	16.40 ± 0.00 ^ab^
CAA (%)	30	18.53 ± 2.22 ^bc^	10.83 ± 1.16 ^def^	23.64 ± 2.62 ^ab^
	60	5.63 ± 0.92 ^g^	13.19 ± 2.22 ^def^	6.30 ± 0.25 ^fg^
	120	8.97 ± 1.24 ^efg^	20.74 ± 1.19 ^abc^	4.13 ± 0.25 ^g^
	180	12.70 ± 1.40 ^de^	24.24 ± 3.76 ^a^	15.49 ± 1.78 ^cd^

Data are expressed as means ± standard deviation (*n* = 3). ^a–g^ Significant different means are labeled with different letters in each column of the graph evaluated by Tukey’s post-hoc test with 95% confidence compared to all types of cleavage (enzymes and times). DPPH: 2,2-Diphenyl-1-picrylhydrazyl, ORAC: oxygen radical absorbance capacity, OH: hydroxyl radical, and CAA: cellular antioxidant activity.

**Table 4:** Photoaging assay represented by the type I collagen concentration (µg/mL) and reactive oxygen species (ROS) production resulting from the preventive assay using selenized chickpea protein hydrolysates (3.125 µg/mL) in HDFa cells 6 h before UVA radiation.

Samples	Type I Collagen (µg/mL)	ROS Production (* RLU × 10^5^)
Control	0.78 ± 0.29 ^b^	20.2 ± 1.43 ^a^
Total protein hydrolysate	0.60 ± 0.22 ^b^	18.0 ± 2.64 ^a^
Protein hydrolysate <10 kDa	3.13 ± 0.57 ^a^	20.3 ± 1.00 ^a^
Protein hydrolysate >10 kDa	0.85 ± 0.32 ^b^	18.4 ± 1.45 ^a^
SeMet	4.88 ± 0.50 ^a^	9.77 ± 0.59 ^b^
MeSeCys	4.73 ± 0.64 ^a^	9.40 ± 0.34 ^b^

Preventive assay: applying hydrolysates and standards 6 h before radiation. Significant differences (*p* < 0.05) were evaluated by Tukey’s multiple comparison tests between all samples and concentrations. ^a,b^ Significant different means are labeled with different letters in each column of the table. * RLU: relative light units of fluorescence. SeMet: selenomethionine, MetSeCys: methyl selenocysteine.

## Data Availability

The data are available on request from the corresponding author.

## Supplementary Materials

The following supporting information can be downloaded at: https://www.mdpi.com/article/10.3390/molecules28083402/s1, Table S1: Cellular antioxidant activity in HDFa cells, inhibition of elastase, and inhibition of collagenase in a range of concentrations from 25 to 3.12 µg/mL of sample.

## Sample Availability

Some of the samples compounds are available from the authors.
