# Supplementary material for: Selenized Chickpea Sprouts Hydrolysates as a Potential Anti-Aging Ingredient
Source: Molecules. 2023 Apr 12;28(8):3402. doi: 10.3390/molecules28083402 (PMC10145560; doi:10.3390/molecules28083402)
Supplement: Supplementary file 1 [file molecules-28-03402-s001.zip › molecules-2332414-supplementary.pdf]

TRIPSIN

Time (min)	R1	R2	Mean	SD	CV (%)
30	14.1	8.9	11.50	3.62	31.50
60	14.1	12.1	13.10	1.36	10.35
120	14.1	13.0	13.52	0.77	5.68
280	14.1	14.9	14.48	0.59	4.06

ALCALASE

Time (min)	R1	R2	Mean	SD	CV (%)
30	16.3	12.3	14.29	2.81	19.69
60	17.0	17.4	17.18	0.26	1.49
120	17.7	19.5	18.63	1.28	6.87
280	17.7	19.5	18.63	1.28	6.87

PEPSIN

Time (min)	R1	R2	Mean	SD	CV (%)
30	34.4	28.2	31.30	4.43	14.14
60	37.6	29.4	33.49	5.75	17.18
120	37.6	34.7	36.12	2.04	5.65
280	37.6	36.7	37.12	0.63	1.69
